# Immune suppression, HLA-risk and potential future immune based targets of oral leukoplakia progression

**DOI:** 10.3389/fdmed.2026.1665195

**Published:** 2026-03-31

**Authors:** Mehar Soni, Bryce DeSantis, Alessandro Villa, Malek Kamoun, James Gates

**Affiliations:** 1 School of Dental Medicine, University of Pennsylvania, Philadelphia, PA, United States; 2 Oral Medicine and Oral Oncology, Miami Cancer Institute, Miami, FL, United States; 3 Pathology and Laboratory Medicine, Hospital of the University of Pennsylvania, Philadelphia, PA, United States; 4 Department of Oral and Maxillofacial Surgery, Penn Dental Medicine, University of Pennsylvania and Hospital of the University of Pennsylvania, Philadelphia, PA, United States

**Keywords:** biomarkers, dysplasia, human leukocyte antigen, leukoplakia, oncology

## Abstract

**Background:**

Oral leukoplakia (OL) is the most common oral potentially malignant disorder and carries a non-trivial risk of malignant transformation. Biomarkers are sought to aid in identifying lesions with high risk for malignant transformation, yet none are currently validated. Recent success of immune based therapies in head and neck oncology highlights immune involvement in the progression of these diseases. The differential expression of classical and non-classical HLA molecules in oral leukoplakia are thought to contribute to their malignant transformation. To better evaluate the role HLA plays in oral pre-malignant lesions, a scoping review was performed.

**Methods:**

A scoping review was conducted in accordance with the PRISMA guidelines across PubMed, EMBASE, and Web of Science. 293 studies were reviewed from 1989 to 2025.

**Results:**

15 studies were screened by title, then 5 original research abstracts were included. Samples studied ranged from *n* = 16 to *n* = 100. It was found that leukoplakia lesions had higher expressions of HLA-E, HLA-G, CircHLA-C, and HLA-DR.

**Conclusion:**

The upregulation of non-classical HLA molecules, particularly HLA-G, HLA-E, and circHLA-C in oral leukoplakia, suggests an immunologic shift, potentially facilitating tumor immune evasion in oral leukoplakic lesions. HLA expression may serve as a potential biomarker for identifying pre-malignant oral lesions at increased risk of malignant transformation. Also, circHLA-C may provide a target for therapeutic intervention in high-risk oral premalignant lesions.

## 1 Introduction

Oral leukoplakia (OL) is defined by the World Health Organization as a predominantly white plaque of questionable risk having excluded other known diseases or disorders that carry no increased risk for cancer (1). OL is classified within the broader group of oral potentially malignant disorders (OPMDs), which encompass a spectrum of clinically and histopathologically diverse lesions with varying risks of malignant transformation. With a prevalence of 1.39% worldwide, the most common type of OPMD is oral leukoplakia (2). To date, the natural history of OL progression is poorly understood. This is the focus of a large area of research with the aim to better stratify risk in oral lesions. The grade of dysplasia as mild, moderate or severe, is currently used to assess risk of malignant transformation but is not completely predictive (3). Additional biomarkers are sought to aid in identifying lesions with high risk for malignant transformation. The immune system is known to play a role in the progression of OL. Recent single cell RNA sequencing data of progressing oral dysplastic lesions show expansion of specific immune cells thought to contribute to inhibition of the innate immune surveillance (4). Several myeloid cell subclusters were found to be expanded in this process, including dendritic cells that express HLA-DR (5). Presence of HLA-DR is known to correlate with increased risk of autoimmune diseases. Less is known about it, or other associated differential HLA expression and oral cancer risk.

The evasion of immune surveillance plays a key role in oncogenesis. Normal immune surveillance involves antigen presentation by human leukocyte antigen (HLA) molecules to T cells to initiate immune responses. Dysregulation of this process is thought to contribute to development of cancer (6). HLA is comprised of classical class I and class II molecules, and non-classical molecules such as HLA-E and G. HLA Class I molecules (HLA-A,B,C) are primarily involved in presenting various antigens such as viral proteins or mutated proteins in cancerous cells to CD8+ cytotoxic T cells for destruction. HLA Class II molecules (DR, DQ, DP) present antigens to CD4+ helper T cells, which play a critical role in coordinating the immune response. Non-classical HLA-E and HLA-G are involved in maintaining immune tolerance and modulating immune responses in specific contexts by interacting with specific inhibitory receptors found on NK cells, T cells, and other immune cells. By binding these receptors, HLA-E and HLA-G inhibit immune responses (7). The dysregulation of HLA expression has been reported in cancer and precancerous conditions (8). For example, overexpression or dysregulation of non-classical HLA molecules such as HLA-G and HLA-E has been correlated with the establishment of an immunosuppressive microenvironment and thus immune evasion (9, 10). This could represent a potential marker for a developing malignancy.

Immunologic changes in the microenvironment of oral leukoplakia are thought to precede malignant transformation (8, 9). RNA sequencing studies show expansion of immune suppressive T cells and other inhibitory clusters that create a permissive environment for unchecked epithelial cell proliferation (11, 12). Additional studies show mechanisms of T cell sequestration away from sites of tumor formation, functionally impairing their ability to detect and kill oral precancer and cancerous cells (13). It is difficult to quantify and use individual cell types or populations as biomarkers given challenges in quantifying and comparing their absolute amounts across slides and patients. It is thought that certain types of HLA expression, or loss of normal Class I MHC molecules may confer risk of immune dysregulation and thus cancer. Studies have shown that increased expression of non-classical HLA molecules, such as HLA-G and HLA-E, are present in oral leukoplakia. Moreover, research of HLA-G and HLA-E shows inhibition of cytotoxic T cell and Natural Killer (NK) cell activity, potentially suppressing anti-tumor immunity (14, 15). HLA-G and HLA-E are also associated with the secretion of immunosuppressive cytokines, such as IL-10 and IFN-γ, which are known to potentiate immune evasion in oral leukoplakia (9, 10). This suggests that nonclassical HLA may play a role in progressing oral leukoplakia to malignancy.

For this reason, we have completed a scoping literature review to further examine the role HLA may play in the malignant transformation of oral leukoplakia. We studied manuscripts that describe oral leukoplakia/premalignant lesions and risk of oral cancer development based upon HLA expression. The main exposure being investigated is expression of HLA subtypes and risk of lesion progression to oral cancer. The comparison is different HLA subtypes (Class I vs. Class II vs. non classical HLA subtype) expressed in oral premalignant lesions to assess the outcome of malignant transformation of oral leukoplakia.

## 2 Methods

**Figure 1:**
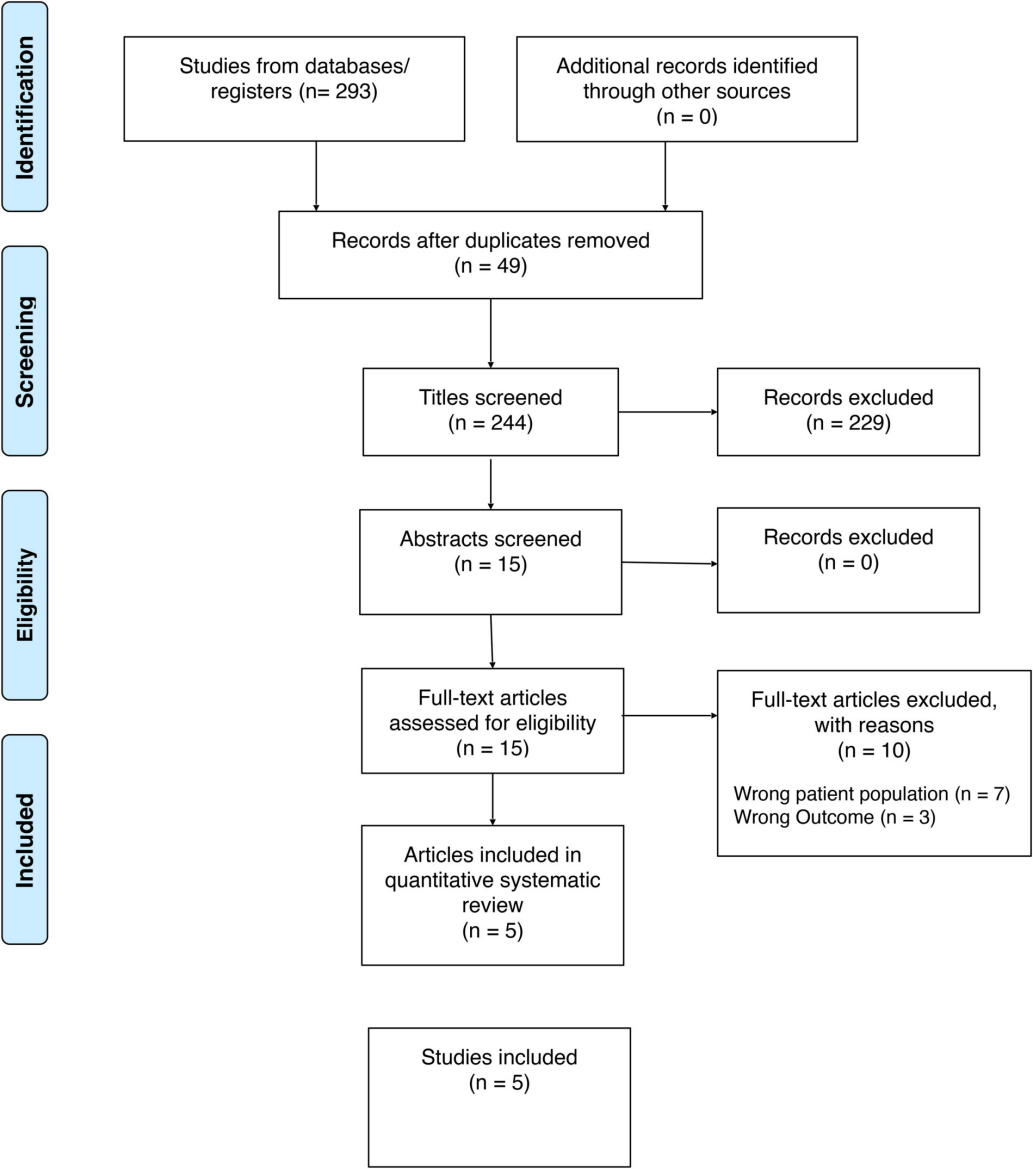
PRISMA flowchart demonstrating scoping review methods.

We conducted a scoping review following the PRISMA (preferred reporting items of systematic reviews and meta-analyses) guidelines as shown in Figure 1. We included all studies on oral leukoplakia and HLA from 1989 to 2025. The quality of studies was evaluated based on their type and relevance to the topic. No study type was excluded or prioritized. The review was conducted across three databases, including PubMed, EMBASE, and Web of Science. Search strategy for PubMed (MEDLINE) was [“Leukoplakia, Oral”(Mesh) OR leukoplakia OR pre-malignant OR pre-cancerous] AND [“Interleukin-10”(Mesh) OR CD8 OR CD 57 OR CD68 OR HLA-G OR HLA-E OR CD68 OR IL-10], and for EMBASE and web of science (“Leukoplakia, Oral” OR leukoplakia OR pre-malignant OR pre-cancerous) AND (“Interleukin-10” OR CD8 OR CD 57 OR CD68 OR HLA-G OR HLA-E OR CD68 OR IL-10). Exclusion criteria were as follows: animal studies, Epstein–Barr Virus, Lupus erythematosus, Human Papillomavirus, oral psoriasis, congenital dyskeratosis, Human immunodeficiency virus, and tobacco. Tobacco exposure was excluded to reduce confounding, as tobacco-associated leukoplakia demonstrates distinct molecular, inflammatory, and immunologic profiles compared to idiopathic OL. Exclusion of tobacco-related lesions allowed for a more focused assessment of HLA-mediated immune mechanisms independent of a well-established carcinogenic exposure. Studies were screened by title to assess for retrieval (*n* = 15), then full abstracts were read to determine final eligibility (*n* = 5). Studies were excluded by population (see exclusion criteria), and condition of interest (premalignant vs. malignant oral lesions).

## 3 Results

Upon applying the search criteria to PubMed, EMBASE, and Web of Science, 293 studies were reviewed on Covidence. After using the appropriate exclusion criteria, five studies were evaluated for the purpose of this scoping review. The five studies included in this scoping review varied in study design, sample size, tissue source, and analytic methodology, but all evaluated the relationship between HLA-related markers and oral leukoplakia or its progression toward malignancy (Table 1).

**Table 1:** Summary of scoping review findings.

Author (first)	Year	Sample type	Control	HLA subtype	*N*=	Method of analysis	Findings
Goncalves	2016	lateral border of tongue adjacent to oral pigmentation	NOM	HLA-E/G	80 (Positive cases *n* = 20)	IHC	OL lesions had higher expressions of HLA-E and HLA-G
Fregonezi	2005	formalin-fixed and paraffin-embedded oral biopsies	N/A	HLA-G	17	IHC	High levels of HLA-G were found in benign (30.6 ± 5.274) and dysplastic lesions (18.6 ± 7.189), while low levels were found in malignant lesions (20.51 ± 5.555). These results were statistically significant (*p* < 0.05)
Yang	2023	OL samples	NOM	circHLA-C	49 (positive *n* = 20)	PCR	High levels of circHLA-C were detected in OL (logFC of OL vs. normal tissue = 7.719, *p* ≤ 0.001)
Xu	2020	OL samples from dorsal/ventral surfaces of tongue	NOM	circHLA-C	26 (positive *n* = 6)	PCR	High levels of circHLA-C were detected in OL (logFC of OL vs. normal tissue = 7.7187, *p* ≤ 0.05)
Bondad-Palmario	1994	Samples from buccal mucosa, lip, tongue, palate, floor of mouth and gingiva	N/A	DR	100	IHC	Elevated levels of HLA-DR were found in moderate and severe dysplastic lesions

IHC, immunohistochemistry; PCR, polymerase chain reaction; OL, oral leukoplakia; NOM, normal oral mucosa.

Goncalves et al. performed immunohistochemical (IHC) analysis for HLA-E and HLA-G in OL, and staining was noted in both cytoplasm and cell membranes (Table 1). Patients with OL underwent an incisional biopsy between 2011 and 2016, and a total of 80 samples were collected. The control group (*n* = 20) consisted of saliva from healthy individuals and tissue from clinically and histologically normal oral mucosa obtained from biopsies of gingival mucosa. Two oral pathologists independently graded epithelial dysplasia based on the criteria described by the WHO and by the Kujan et al. binary system (high/low-risk of malignant transformation). Samples showed the following distribution: severe dysplasia (*n* = 15), moderate (*n* = 20), mild (*n* = 32), and no dysplasia (*n* = 13), with 18 samples with high-risk and 62 samples with low-risk of malignant transformation.

HLA- G and E were evaluated in epithelial cells (keratinocytes) and adjacent connective tissue cells (immune-inflammatory, endothelial cells, and fibroblasts). Strength of staining was assessed by calculating an immunoreactive score. Of note, lesions that had higher expressions of HLA-E and HLA-G by IHC had a higher incidence of malignant transformation compared to the controls. This finding was independent of the degree of dysplasia. An additional significant finding is that the expression of HLA-G and E in oral leukoplakia correlated with the levels of expression seen in the OSCC group (8). The study is limited by its retrospective design and lack of longitudinal follow-up, which prevents direct evaluation of malignant transformation over time. Additionally, while the sample size was relatively large compared with other studies in this review, the use of immunohistochemical scoring introduces potential observer variability despite the use of standardized scoring methods.

Similarly, Fregonezi et al. collected oral biopsies (benign, oral leukoplakia, and oral squamous cell carcinomas) and analyzed them for anti-HLA-G antibodies (16). 51 formalin-fixed and paraffin-embedded oral biopsies from archives between 1992 and 2005 were used. The biopsies were stratified into three groups as follows: oral benign lesions (oral hyperplasia and papilloma, *n* = 16), oral premalignant lesions (oral leukoplakia with dysplasia and lichen planus, *n* = 17), and malignant lesions (oral squamous cell carcinoma, *n* = 18). Tumor grades were evaluated histologically and IHC was performed using the streptavidin-biotin system for the detection of HLA-G antigens (Table 1). The results were classified as negative when the cytoplasm was immunolabelling (≤25%), and positive (immunolabeling <25% until 100%). The positive control was a section of trophoblastic tissue from a third-trimester human placenta, while the negative control was the same human tissue used as positive control, in which the primary antibody was omitted from the assay. High levels of HLA-G (30.6 ± 5.274) were found in benign and (18.6 ± 7.189) premalignant oral lesions. In contrast, low levels of HLA-G (20.51 ± 5.555) were observed in malignant oral lesions. Statistical significance was observed between high expression of HLA-G in benign vs. premalignant and malignant oral lesions (*p* < 0.05). Interpretation of these findings is limited by the relatively small sample size and the grouping of different premalignant conditions within the same category. Additionally, the cross-sectional design and lack of long-term follow-up prevents determination of whether lesions with higher HLA-G expression ultimately progressed to carcinoma.

To investigate the potential role circRNAs might in the pathogenesis of OL and Oral lichen planus (OLP), Yang et al. examined their differential expression as compared to normal and cancerous tissues (Table 1). They utilized high throughput sequencing technology to determine which circRNAs were enriched in these diseases as compared to normal tissue and confirmed the findings through PCR analysis. Five circRNAs were highly expressed in OL, of which circHLA-C was found to have the most elevated expression on sequencing. In addition, gene ontology functional analysis showed increased activity of biologic pathways associated with immune processes, indicating its potential marker of OL development, and its distinction from normal mucosa and possible marker of early detection for oral cancer (17). However, the study relied primarily on sequencing-based discovery methods, limiting conclusions regarding the clinical applicability of circHLA-C as a biomarker.

In a similar study, Xu et al. studied six OL tissue samples were obtained from two male and four female patients, compared to 6 samples or normal oral mucosa from 2018 (Table 1). Based on the diagnostic criteria of WHO, the patients were diagnosed by histopathological examination. Xu et al. revealed that the most upregulated circRNA in oral leukoplakia was circHLA-C (18). The diagnostic potential of circHLA-C was evaluated using qRT-PCR, yielding an area under the curve (AUC) of 0.955. A statistically significant correlation was observed between circHLA-C expression levels and the degree of dysplasia, suggesting its potential role as a biomarker for oral leukoplakia progression (18). Despite these promising findings, the small sample size and study design significantly limit the generalizability of the results, highlighting the need for larger studies to confirm the diagnostic and prognostic value of circHLA-C.

Lastly, Bondad-Palmario used 100 samples of oral leukoplakia histologically graded based on WHO criteria. Immunohistochemical controls consisted of the replacement of primary antibodies with normal or blocking serum. All specimens that reacted with HLA-DR antibody gave positive results, which were especially strong in Langerhans cells. As the expression of HLA-DR was widely distributed on numerous different cell types, the cell count was not determined. Bondad-Palmario did note that HLA-DR expression was increased in keratinocytes in severely dysplastic oral leukoplakia lesions (19). However, because HLA-DR is widely expressed among various immune and epithelial cell types, quantitative cell counting and analysis was not performed, limiting the ability to compare expression levels between lesions. In addition, the study lacked a control group of normal mucosa or long-term clinical follow-up, making it difficult to determine whether increased HLA-DR expression is predictive of malignant transformation or simply reflective of local immune activation.

Collectively, these studies suggest that alterations in HLA-related molecules and associated regulatory pathways may contribute to immune modulation in oral leukoplakia and its potential progression toward malignancy. However, the existing literature is limited by sample size, heterogeneous methodologies, and retrospective or cross-sectional designs. The lack of longitudinal studies evaluating the predictive value of these markers for malignant transformation represents a significant gap in the current evidence base. Future research incorporating larger cohorts and prospective follow-up will be necessary to determine whether HLA-related markers can serve as reliable biomarkers for early detection or risk stratification in oral leukoplakia.

## 4 Discussion

Evasion of the host immune system is regarded as a hallmark of cancer development. It is thought that HLA may play a role in tumor immune response via antigen presentation and activation of innate and adaptive immunity for cancer cell destruction, and its dysfunction may permit epithelial cell proliferation (20). As so, the potential role of HLA expression in oncogenesis remains a topic of interest and was the reason for this investigation. In this review, Goncalves and Fregonezi et al. showed that HLA-G expression was increased in OL. Goncalves et al. found the same to be true for HLA-E, which was not studied by Fregonezi's group. In addition, Goncalves et al. found increased HLA-E and G expression to predict malignant transformation, independent of the degree of histologic dysplasia. Investigation into HLA risks of OL by Bondard-Palmario found increased HLA-DR expression to be present in severe dysplasia. Yang et al. and Xu et al. found that circHLA-C expression is upregulated in oral leukoplakia compared to normal mucosa, and correlates to dysplasia severity (17, 18). This is corroborated by Momen-Heravi et al.'s work implicating circHLA-C in oral cancer initiation and progression. They detail the function of circHLA-C in oral cancer and as it relates to potential use as a diagnostic tool and target for therapy (21). Thus, in this literature review we found that increased HLA-E, G, circHLA-C, and DR expression was present in progressing OL. This furthers the idea that expression of non-classical HLA molecules in premalignant lesions such as OL may permit evasion of immune surveillance as an early event in oncogenesis and could serve as a biomarker for progression.

Recently, circRNA has become a therapeutic target of interest for cancers of other types and autoimmune diseases (22). Loss-of-function approaches include the use of RNA interference (RNAi) mechanisms (23). These short dsRNA molecules target and bind the circRNA, silencing their effect and marking them for cleavage and destruction (24). Challenges with (RNAi) include their rapid degradation, difficulty with delivery and side effects. These studies provide intrigue into the potential future targetability of circRNAs in diseases which overexpress them, such as that of OL and the overexpression of circ-HLA-C.

Inherent to the success of immune-based therapies for oral cancer and precancer is the implication of its role in cancer initiation and progression (25, 26). While research examines the cellular landscape of this immune contribution, less investigation has been completed to detect the systems and cellular programs that regulate these cells (5, 9, 27). In addition, it is well recognized that multiple different immunologic phenotypes of OL exist, which complicates prognosticating lesion progression based upon cell types alone (28–30). This is the impetus for our investigation; to research a gap in the literature regarding what master regulators might exist in the host immune system that causes susceptibility to OL progression and malignant transformation. Given the known risk and susceptibility that expression of certain HLA molecules portends on autoimmune diseases, we endeavored on this investigation in oral cancer, despite and because of the paucity in literature. The limited number of studies and data to review in our investigation provides us the ability to describe our findings, but statistical analysis is not possible. This is the most important limitation to this review; however, we see it justified given the critical gap in our understanding of immunologic control mechanisms that lead to oral cancer. Additional limitations of our review include the retrospective nature of the investigations, with inherent recall and selection bias. In addition there was significant heterogeneity in study design and methods which make it difficult to draw conclusions.

However, this review furthers the concept of immune mediated progression and transformation of oral premalignant lesions such as OL to oral cancer. Additional profiling of both classical and non-classical HLA molecules in oral leukoplakia may provide valuable insights into the mechanisms underlying the progression of oral leukoplakia to oral squamous cell carcinoma. HLA-G and HLA-E may function as biomarkers for high-risk oral leukoplakia exhibiting immune evasion. CircHLA-C may serve as both a potential biomarker and therapeutic target for OL. This review should serve as the basis for additional prospective investigation into potential diagnostic and therapeutic applications of HLA for oral leukoplakia and cancer.

## Data Availability

The original contributions presented in the study are included in the article/Supplementary Material, further inquiries can be directed to the corresponding author.
